# SKOPE-IT (Shareable Knowledge Objects as Portable Intelligent Tutors): overlaying natural language tutoring on an adaptive learning system for mathematics

**DOI:** 10.1186/s40594-018-0109-4

**Published:** 2018-04-13

**Authors:** Benjamin D. Nye, Philip I. Pavlik, Alistair Windsor, Andrew M. Olney, Mustafa Hajeer, Xiangen Hu

**Affiliations:** 1 Institute for Creative Technologies, University of Southern California, 12015 Waterfront Dr., Playa Vista, 90012 CA USA; 2 Institute for Intelligent Systems, University of Memphis, 365 Innovation Dr., Memphis, 38152 TN USA

**Keywords:** Intelligent tutoring systems, Natural language tutoring, Mathematics education, Worked examples, Isomorphic examples, System integration

## Abstract

**Background:**

This study investigated learning outcomes and user perceptions from interactions with a hybrid intelligent tutoring system created by combining the AutoTutor conversational tutoring system with the Assessment and Learning in Knowledge Spaces (ALEKS) adaptive learning system for mathematics. This hybrid intelligent tutoring system (ITS) uses a service-oriented architecture to combine these two web-based systems. Self-explanation tutoring dialogs were used to talk students through step-by-step worked examples to algebra problems. These worked examples presented an isomorphic problem to the preceding algebra problem that the student could not solve in the adaptive learning system.

**Results:**

Due to crossover issues between conditions, experimental versus control condition assignment did not show significant differences in learning gains. However, strong dose-dependent learning gains were observed that could not be otherwise explained by either initial mastery or time-on-task. User perceptions of the dialog-based tutoring were mixed, and survey results indicate that this may be due to the pacing of dialog-based tutoring using voice, students judging the agents based on their own performance (i.e., the quality of their answers to agent questions), and the students’ expectations about mathematics pedagogy (i.e., expecting to solving problems rather than talking about concepts). Across all users, learning was most strongly influenced by time spent studying, which correlated with students’ self-reported tendencies toward effort avoidance, effective study habits, and beliefs about their ability to improve in mathematics with effort.

**Conclusions:**

Integrating multiple adaptive tutoring systems with complementary strengths shows some potential to improve learning. However, managing learner expectations during transitions between systems remains an open research area. Finally, while personalized adaptation can improve learning efficiency, effort and time-on-task for learning remains a dominant factor that must be considered by interventions.

## Overview

Scaling up intelligent tutoring systems (ITS) to mainstream educational contexts has been a significant challenge for the research community. While ITS have shown significant learning gains over traditional educational technology (VanLehn 2011; Kulik and Fletcher 2016), the cost to develop and distribute ITS interventions has often meant that less adaptive technologies dominate the growing educational technology landscape (e.g., web-based homework, online videos). Because of this, a likely future for intelligent tutoring systems is to integrate and interoperate with a variety of other systems, some of which may be adaptive while others mainly present static content (e.g., traditional learning management systems).

Scaling challenges disproportionately impact ITS design. Handling a large range of domains is difficult for tutoring systems because ITS tend to be tightly linked to specific learning activities (e.g., solving a math problem, drawing a diagram, paraphrasing an essay). Expanding these ITS to a new activity often requires significant additions to ITS modules that handle assessment (e.g., evaluating domain-specific task performance) and communication (e.g., providing feedback on the task). As such, coverage can be an issue and overall learning gains from ITS tend to be higher on locally developed tests rather than standardized tests (Kulik and Fletcher 2016). Given the effort needed to extend an ITS to a new learning task, an ITS intended for a wide range of domains needs to focus on relatively universal interactive tasks.

Two common learning tasks are step-by-step problem solving and natural language conversation, which have each been used for a number of domains (Nye et al. 2013). However, it is non-trivial to combine these tasks due to the difficulty of building conversations that can account for changes in the problem-solving state. As an alternative to trying to maintain a coherent conversation about a problem that a learner is constantly changing, one possible solution is to align at the problem level, such that the tutorial conversations address the same skills and knowledge that the problem-solving steps require. This abstraction potentially allows conversational tutoring to be overlaid on any learning activity, without needing to develop an exhaustive 1-to-1 mapping of tutorial dialog to address the entire problem-solving state space. Instead, tutorial dialogs align to a single path through that space, enabling a coherent conversation about the solution path and its steps for a particular example. This is similar to example-tracing tutors, which help tutor solving the steps of a specific problem (Aleven et al. 2009), except in this case the goal is not to solve the steps but to explain why and how they were solved that way. Such self-explanations are intended to highlight the generalizable skills that should transfer to a variety of math contexts rather than only reviewing the specific procedures for that problem.

To implement this approach, we have developed a method for annotating an HTML page with tutoring conversations that sequentially discuss the concepts associated with each part of the page. In the present study, we used this method to add tutoring dialogs to the problem steps of worked examples of algebra problems. While this study applies this approach to mathematics, it could also be used for a variety of domains, such as reading comprehension (e.g., dialogs could be associated with positions in a news article), tutoring the steps to a how-to manual, or promoting reflection about a decision-making scenario.

The above integration strategy presents a major methodological challenge in that standalone intelligent tutoring systems have traditionally been “application islands” that cannot easily interact with other ITS and learning platforms (Roschelle and Kaput 1996). However, the emerging ecosystem of web applications depends on integrating web services hosted across multiple domains and managed by different institutions. As a field, we need to refine methodologies to integrate different learning technologies (i.e., hybrid ITS). Our approach to this challenge was to build and apply a web service-oriented architecture approach to combine components. Using this framework, we integrated our conversational tutoring system into an existing commercial adaptive learning system. Based on reactions by different stakeholders (e.g., teachers, students), we identified a number of advantages for this kind of multi-system integration (e.g., combining complementary strengths) and disadvantages (e.g., pacing differences and student confusion over the role of different systems) that will be discussed in this paper.

The Shareable Knowledge Objects as Portable Intelligent Tutors (SKOPE-IT) system described in this paper pushes the boundaries of domain-agnostic tutoring by annotating where natural language-tutoring dialogs occur in an HTML page (e.g., a math worked example, a how-to page, etc.). SKOPE-IT stores and delivers web-based conversational tutoring that can be integrated as a real-time web service and easily embedded into existing web applications. SKOPE-IT is also designed to integrate multiple web applications using semantic messaging. In this study, it was used to integrate the AutoTutor Conversation Engine (ACE; Nye et al. 2014b), the ALEKS (Assessment and Learning in Knowledge Spaces) commercial mathematics learning system (Falmagne et al. 2013), and a number of domain-specific services designed to address some distinct challenges for natural language dialogs about algebra. Evaluation outcomes for the first evaluation of this system are presented, as well as lessons learned for building future hybrid systems using conversational tutoring.

## Motivation to integrate: complementary strengths

Future intelligent tutoring systems (ITS) will need to integrate with other learning systems, particularly other intelligent systems. AutoTutor and ALEKS were integrated using the SKOPE-IT system due to their complementary strengths. While AutoTutor and ALEKS are both adaptive learning systems, they fall on very different ends of the spectrum with respect to adaptivity: ALEKS works on the outer-loop (problem selection), while AutoTutor adapts at the inner-loop (problem-step level) during a problem (VanLehn 2006).

ALEKS is a commercial online learning environment with material in a number of domains including mathematics, chemistry, and business (Falmagne et al. 2013). ALEKS is only adaptive at the outer-loop, where it enforces mastery learning: students are only able to practice problems after they have mastered all of the required prerequisites (i.e., macro-adaptive mastery learning). This prerequisite structure is modeled using knowledge space theory, which assumes that certain sets of knowledge components (i.e., knowledge spaces) can only be mastered after a certain subset of other components are mastered (Falmagne et al. 2013). In practice, for ALEKS algebra, this can be represented using a directed acyclic graph, where only the boundary of knowledge components is available to be learned at any time.

**Fig. 1:**
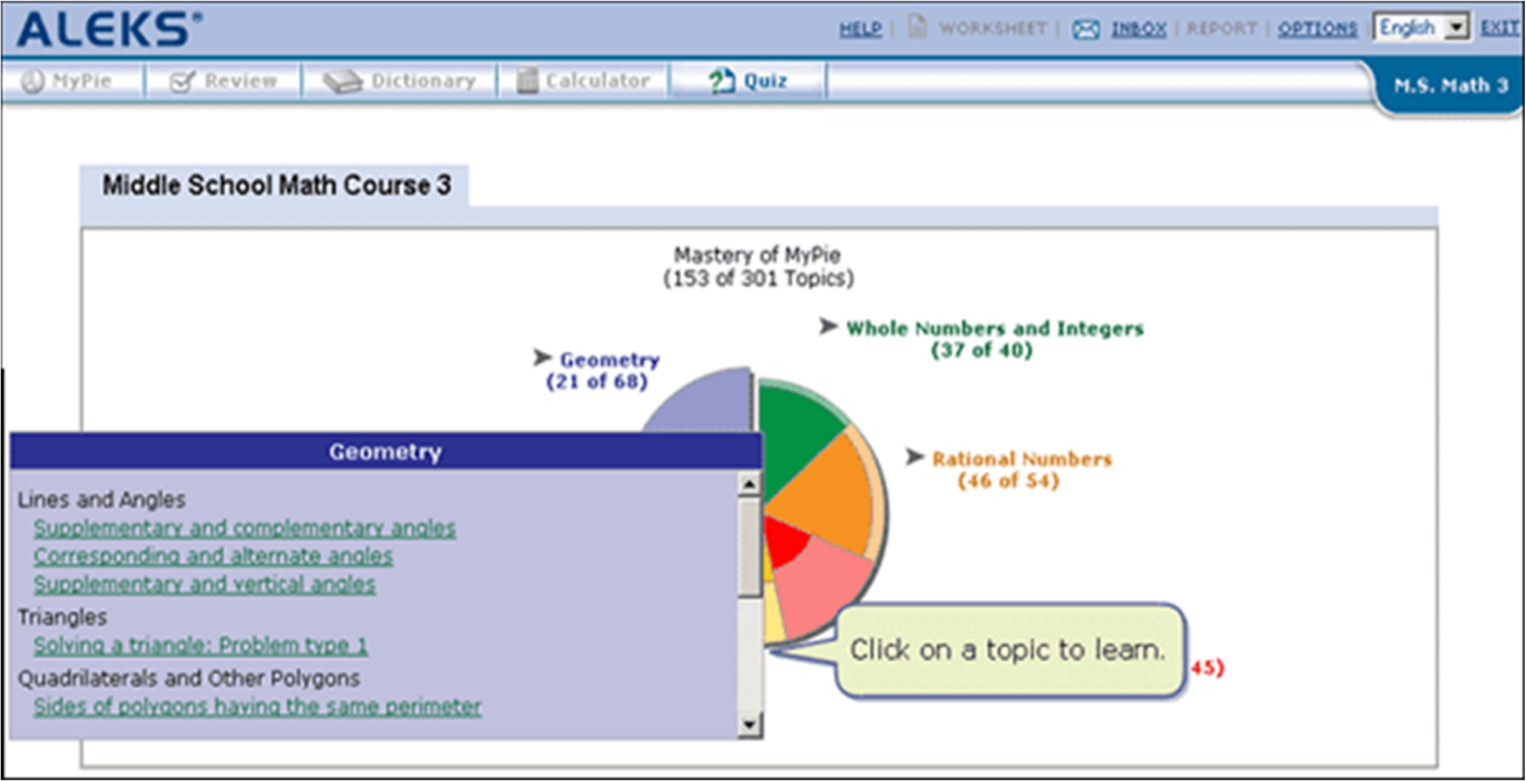
ALEKS pie: adaptive availability of skills (KC’s) to master

**Fig. 2:**
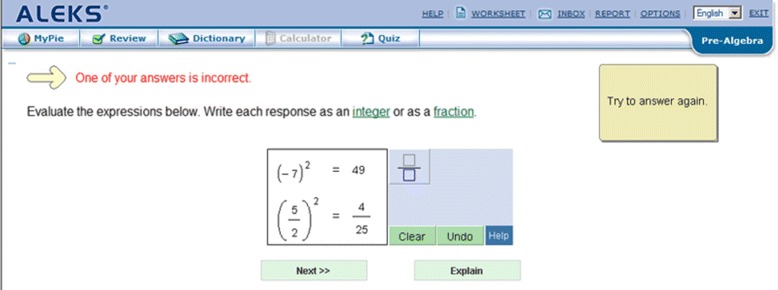
ALEKS problem: attempting a problem, with options to submit (“Next”) or “Explain”

**Fig. 3:**
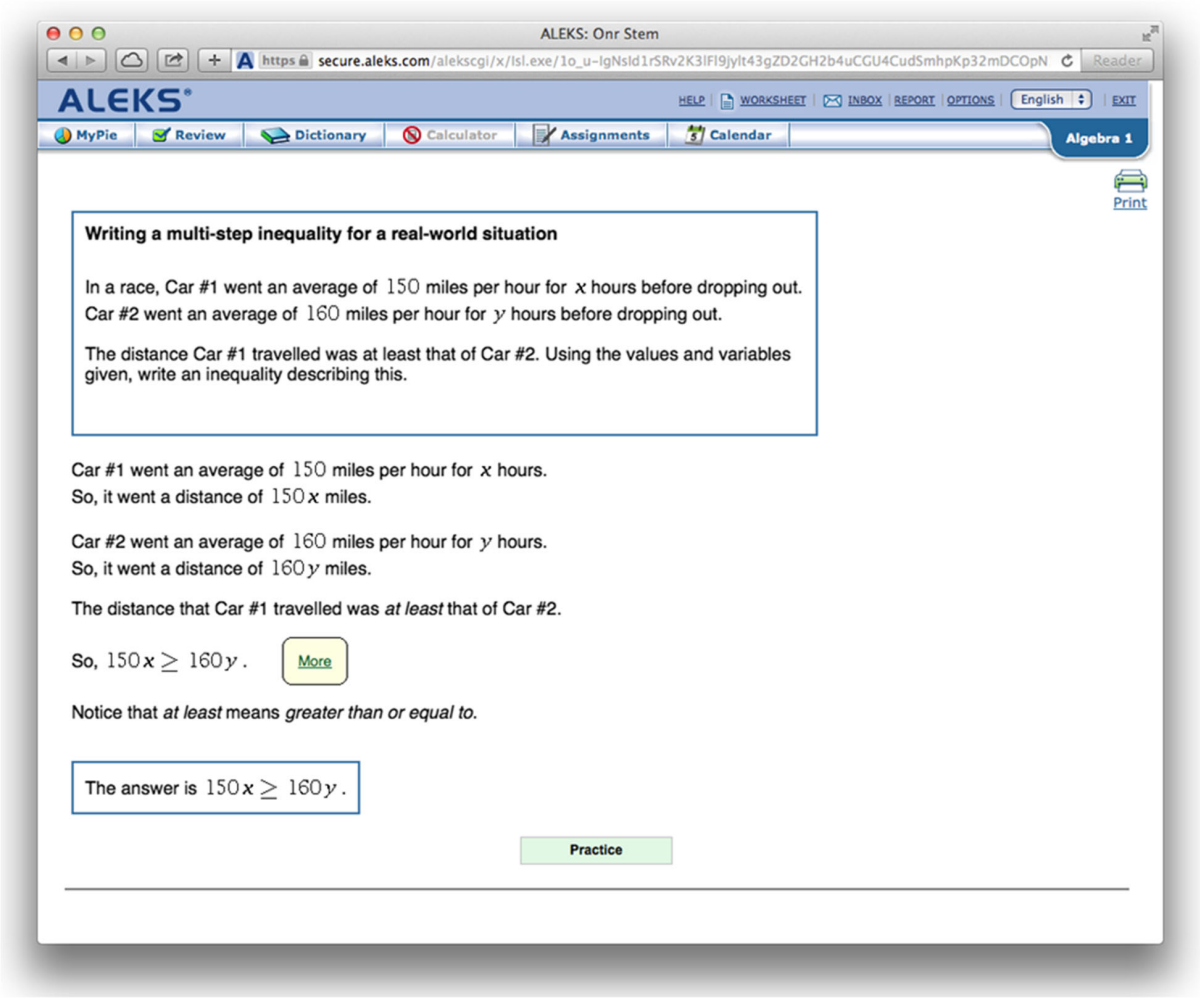
ALEKS explanation: a worked example presented by ALEKS

Student interaction in ALEKS centers around selecting a skill to master, which is done by selecting an available skill from a “pie” which shows groups of related skills (shown in Fig. 1). Once a skill is selected, learners complete isomorphic (equivalent structure) practice problems until they can solve that problem type consistently, using an interface similar to Fig. 2. In this scheme, every new category of isomorphic problems requires exactly one more skill when compared to the skills that the learner already knows. When a student cannot solve a problem, they can view a step-by-step worked example by hitting the “Explain” button (shown in Fig. 3). Also, being a commercial system, ALEKS has a range of tools for a teacher to customize course content and view reports on student learning.

Studies on ALEKS have shown fairly consistent evidence that the system improves mathematics skills as measured by standardized tests and by pre-test/post-test designs (Sabo et al. 2013; Craig et al. 2013). Exact effect sizes as compared to different types of control conditions (e.g., non-adaptive online learning, traditional lectures) are still being investigated, but so far have shown comparable outcomes to other instructional approaches that are known to be effective. For example, one study reported that ALEKS in an after-school program offered gains comparable to expert teachers running supplementary classes, but with lower burden on facilitator/teacher time (Craig et al. 2013). A second study reported learning gains comparable to the cognitive tutor (Sabo et al. 2013), which has demonstrated statistically significant gains on large-scale randomized controlled studies (Pane et al. 2014). ALEKS has also shown evidence that it may reduce achievement gaps between white and black students when integrated into instruction that would otherwise not include an online adaptive learning system (Huang et al. 2016).

On the other hand, the AutoTutor Conversation Engine is a web service that drives conversations with one or more conversational agents (Graesser et al. 2012; Nye et al. 2014b). While this system can be integrated into larger ITS that have outer-loop adaptivity, each conversation script focuses on inner-loop adaptivity to the student’s natural language text input. In terms of the Bloom (1956) taxonomy, ALEKS focuses primarily on *applying* algebra skills, while AutoTutor questions can help students *understand*, *analyze*, and *evaluate* algebraic concepts. The goal of a typical AutoTutor conversation is to ask a question and then help the learner express one or more expectations (main ideas), while providing leading questions (hints) and fill-in-the-blank questions (prompts) to help scaffold the learner’s self-explanation (Graesser et al. 2012).

AutoTutor-style tutoring has produced learning gains when compared to control conditions such as reading textbooks or no intervention (averaging about 0.8 *σ*) across a variety of domains, including computer literacy, physics, and scientific methods (Graesser et al. 2012; Nye et al. 2014a). Comparisons against human tutors showed no significant differences in overall learning gains (Vanlehn et al. 2007). A key element of AutoTutor’s effectiveness is that it emulates how human tutors work with students, which revolves around scaffolding students to self-explain key expectations required to solve a problem (Graesser et al. 2012). With that said, the majority of these studies have been done under controlled conditions, rather than in real-life courses where external confounds would impact learning gains. Additionally, like many systems deployed for research, AutoTutor does not yet have extensive interfaces for teacher control and management.

In this project, ALEKS provided a foundation for procedural practice and adaptive practice problem selection, while AutoTutor offered potential learning gains driven by interactive natural language tutoring. The integrated presentation of AutoTutor and ALEKS learning occurred during the “Explain” page for the ALEKS item. The ALEKS Explain page presents a worked example solution to the specific problem that the learner could not solve correctly (e.g., Fig. 3). While the explanation gives the steps of the solution, it explains few of the underlying principles in detail.

**Fig. 4:**
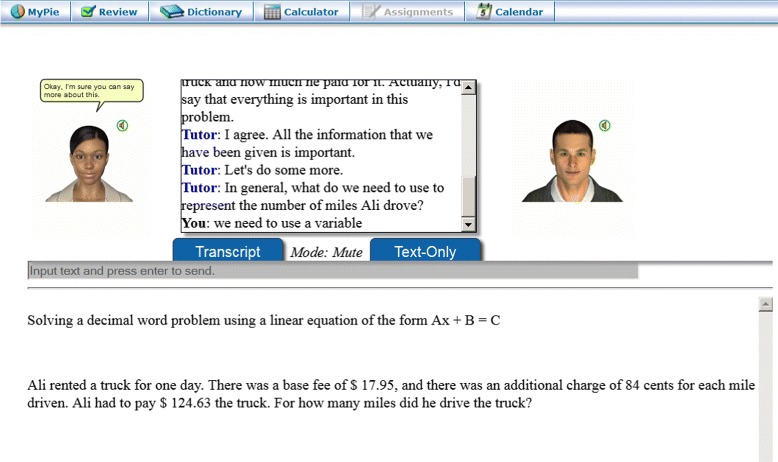
Integration of a tutored worked example into ALEKS

The SKOPE-IT system integrated AutoTutor dialogs by presenting a tutoring-enhanced worked example for an isomorphic problem, with a series of small dialogs that each cover a key principle about that problem type. From the user’s perspective, these small dialogs might appear to be a single longer dialog, but they were functionally independent by design: making each step-specific dialog modular allows for flexibility in disabling or reusing dialogs (e.g., if the same procedure, such as verification, should be applied). The HTML for the worked example (including any images) is dynamically rendered after each dialog finishes and the learner talks about the latest solution step. Figure 4 shows a tutored worked example, which can be compared against the standard ALEKS explanation in Fig. 3. Since the current dialog is related to an early step of the problem, the remainder of the solution is hidden (the blank area at the bottom, which will scroll down as new content appears). The full text of the example shown in Fig. 4 can be found in the Appendix. After completing the worked example with the embedded dialogs, the learner sees the complete worked example from ALEKS for the exact problem that they were unable to solve. This design was based on three learning principles discussed in the next section.

## Theoretical underpinnings

When building SKOPE-IT, the goal was to combine: (1) worked examples (Schwonke et al. 2009), (2) self-explanation (Aleven et al. 2004), and (3) impasse-driven learning (VanLehn et al. 2003).

First, worked examples have been shown to improve conceptual understanding in intelligent tutoring systems (Schwonke et al. 2009) and are complementary to self-explanation tasks (Renkl 2005). Moreover, existing instructional materials in many existing online systems tend to include a wealth of worked examples and solutions, either presented using static multimedia (e.g., WikiHow) or video form (e.g., Khan Academy). Unfortunately, non-interactive media can suffer from shallow attention and processing: in some studies, reading a textbook fails to outperform even do-nothing controls (Vanlehn et al. 2007). The default ALEKS Explain functionality potentially suffers from some of these issues of shallow processing since it does not provide any significant interaction.

Second, natural language tutoring offers a clear complement to static worked examples. Renkl (2005) notes that worked examples are most effective when learners self-explain, generalizable principles are highlighted, structural features and mappings between representations are salient, and the building blocks for a solution are isolated into identifiable steps. Natural language tutoring such as AutoTutor’s expectation coverage dialogs directly prompt the learner to self-explain until certain content coverage criteria are met (Graesser et al. 2012). Moreover, well-designed tutoring dialogs can be used to focus the learner’s explanations toward key concepts and principles, important structural features of the problem, and can also scaffold the learner to map between representations (e.g., formulas to explanations).

The third principle behind this work was to harness impasse-driven learning. ITS interventions such as hints or tutoring dialogs are more likely to promote learning when the student feels stuck or confused (VanLehn et al. 2003). Since students only request an ALEKS explanation when they cannot solve a problem, explanations occur at an impasse. Impasse learning may benefit students since impasses can trigger confusion, which tends to precede learning (Lehman et al. 2012). As such, the tutoring should be more effective at these times.

## SKOPE-IT design

While SKOPE-IT has close ties to AutoTutor and is embedded in ALEKS for the study presented next, the distinct role played by SKOPE-IT was to align dialogs to HTML worked examples using annotations and to coordinate real-time communication between a variety of web services (Nye et al. 2014b). In SKOPE-IT, each service communicates with other services by passing their semantic messages into a gateway node (e.g., a request that the TutorAgent speak some “text”). Gateway nodes determine the network structure by communicating with each other across standardized web protocols. Currently, the main protocols are HTML5 postMessage, which handles cross-domain communication inside webclients, and webSockets, which support bidirectional communication between webclients and servers (i.e., the server can “push” messages to a connected client in real-time). Since messages are handled by services based on the content that they contain, there are no tight connections between services. This makes it straightforward to move functionality between different services, even those services that lie on different servers or are moving from server-side code (e.g., Python, Java) to client-side code (e.g., JavaScript). The messaging architecture for this system has been released and continues to be developed as the SuperGLU (Generalized Learning Utilities) framework (https://github.com/GeneralizedLearningUtilities).

**Fig. 5:**
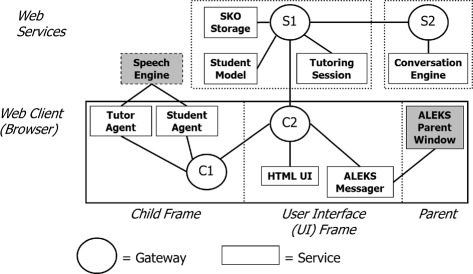
SKOPE-IT core service structure

This architecture lends itself to building a variety of purpose-specific services, rather than a specific implementation of a traditional four-model ITS architecture. Figure 5 shows the services integrated in this project. Gateway nodes are shown as circles, where client gateways and server-side gateways begin with C and S, respectively, (e.g., C1 versus S1). Services are shown as rectangles, with third-party services are shown in gray. These include ALEKS and a commercial Speech Engine service. In this configuration, messages were passed using a fanout policy along the gateway graph (i.e., all services could receive any message). One exception to this scheme was that, in some cases, the the session id was removed from purely server-side messages, which would prevent that message from reaching any client browser (since without the session id, it would then be impossible to identify which user that message was related to). This was done to eliminate unnecessary network traffic.

## Domain content: 50 algebra worked examples

Math is a new domain for AutoTutor, and it has particular challenges for a natural language-tutoring system. Since the role of AutoTutor in this system was to facilitate discussion on the principles and features of an existing problem, it was unnecessary to make the learner write complex math syntax. However, even for simple statements (e.g., “ *x*+*y*”), math relies on variables with little general semantic meaning, making them more difficult to evaluate. Also, when the animated pedagogical agents speak there are challenges with articulating the sometimes ambiguous syntax of formulas.

Three natural language processing enhancements were made so that AutoTutor could handle math dialogs more effectively. First, a corpus of algebra-related documents was collected using webcrawlers and a new math LSA (Latent Semantic Analysis) semantic space was generated (Landauer et al. 1998). This allowed better evaluation of synonyms for math concepts. Second, to understand equivalent words for operators, numbers, and other common terms, equivalency sets were defined (e.g., over a dozen terms were treated as exact matches for “add”, including “+”, “sum”, and “plus”). Using these sets, pre-processing was applied to both the student’s input and also for any keywords that authors required for that dialog, so that any term found in a set was placed in a canonical form before comparing terms.

Third, to speak formulas, a simple parser was designed to convert math formulas into English terms. One complication for this process was that people do not speak formulas precisely, so off-the-shelf libraries produced stilted language, e.g. the formula “z*(*x*+5)” being spoken as “*z* times left parenthesis *x*....” To avoid this, our parser did not translate grouping characters to speech. Instead, a plain-English version of the formula was spoken (e.g., “*z* times *x* plus 5”), while the original exact formula spoken was shown in the chat log so that the precise values were clear (shown in Fig. 4). Another challenge for articulating formulas was the difficulty in disambiguating certain symbols, such as the difference between a function name and a series of multiplied variables (e.g., “tan(*x*)” is “tangent of *x*”, but “an(*x*)” is typically “a n times *x*”). This was handled by checking a table of common functions and constants that were handled before breaking groups down into simpler variables and terms.

The content for the tutored worked examples was based on 50 worked examples drawn from solutions to ALEKS items that were aligned to the Common Core Algebra I curriculum. These 50 items were chosen because they focused on algebra topics with stronger ties to conceptual understanding, such as representation mapping, systems of equations, or problems that included units of measurement. As a result, approximately half of the tutored worked examples involved word problems. These worked examples cover only a fairly small fraction of ALEKS items: as of this study, their algebra I curriculum included 693 item types. Each item type is a generator for multiple isomorphic problems. ALEKS generates isomorphic problems by varying not only the numbers but also the context (e.g., different quantity types in a word problem).

For each worked example, 5–12 brief dialogs were authored (407 dialogs in total). In general, each brief dialog attempted to target a single knowledge component, though a small number of dialogs targeted multiple skills. Examples of knowledge components used by the system would be “IndepDep: Distinguish independent and dependent variables,” “SolveSystemBySubstitution: Solve system by substituting equivalent expression,” or “VerifySolution: Verify a given number is a solution to an equation by substitution.” Each dialog included statements by both a tutor agent and a student agent. Two types of dialogs were authored: trialogs (75% of dialogs) and vicarious tutoring (25% of dialogs). In a trialog, the tutor agent asked a main question and both the human student and student agent would respond with answers, then the tutor agent would provide feedback and follow-up questions that scaffold the explanation. In vicarious tutoring, the peer student agent modeled an explanation with the tutor to demonstrate a concept. Vicarious dialogs were used to explain concepts that were either contained a nuanced skill (e.g., a common but subtle misconception that learners might not articulate), a step that required multiple simultaneous skills (to enable focusing the dialog on one of them), or a concept that might be hard to process using natural language dialog responses. To complete a worked example, the human student needed to complete each of the brief dialogs (one for each key step). These dialogs proceeded shortly after each other, giving the feeling of a longer ongoing conversation. Students also had the option to replay certain dialogs associated with a step by clicking on a special button embedded in that step.

The length of the dialogs depended on the quality of the learner’s input: perfect answers complete the dialog immediately, while incomplete or wrong answers lead to a series of hints and prompts. Depending on the quality of student input and student typing speed, a single dialog can take between 15 s and 3 min. The time for a novice student to carefully complete a tutored worked example (5–12 dialogs) would typically be between 10 and 20 min. For a highly knowledgeable student, the same example would likely be less than 5 min. By comparison, the time to read through an untutored worked example appeared to range between 0 and 10 min, ranging from students who only looked at the right answer (e.g., nearly 0) to students that tried to re-work their own solution to reach the correct answer (up to 10 min).

## Hypotheses

Using this integrated system, a study was conducted to look at three main research questions:

1. *H1*: That dialog-based tutoring enhancements to ALEKS explanations will lead to greater learning gains on ALEKS assessments (near transfer).
2. *H2*: That dialog-based tutoring will lead to higher general math competency, as measured by a far-transfer test.
3. *H3*: That students with stronger beliefs in math concept mastery will be more likely to prefer to work with the agents.

In the following section, the methods and measures to look at these hypotheses are described.

## Study methodology and population

### Design

The experiment was integrated into normal class activities for three sections of a college basic algebra class. Subjects were aware that they were participating in an experiment and would be assigned to one of two conditions. The course combined short lectures with students working problems on the ALEKS system. This course contained a total of 240 ALEKS items, of which 30 items had tutoring-enhanced explanations. A lecturer unaffiliated with the experimenters led all three class sections and was not made aware of the students’ condition assignments (though they could have inferred it from in-class ALEKS use). The duration of the intervention in the class was 12 weeks.

The SKOPE-IT system randomly assigned each student to an experimental condition with tutoring-enhanced items (experimental) or to a control where ALEKS presented its usual non-interactive solutions (control). Due to two issues, dosage was inconsistent across conditions. First, since ALEKS guided problem selection and not all problems had dialogs (only 12.5%), participants in the experimental condition were not expected to receive entirely equal dosage. Second, due to a glitch in authentication, the control condition was presented with tutoring for 3 weeks out of the 12-week course, making the control condition effectively a lower-dose treatment.

### Participants

Three sections of a Mid-South college basic algebra class participated in this study (112 students). Basic algebra is a course for students with very low mathematics placement scores, in that any student with lower placement scores than this population would be required to take remedial mathematics at a 2-year institution instead. Out of 112 students, 9 dropped out in their first weeks of the class before using ALEKS significantly and were excluded from the analysis. The total number of participants in each condition was initially 49 for experimental condition and 63 for the control. After early dropouts, the number of participants fell to 42 experimental and 61 control.

Overall attrition from the class was high: out of 112 students, 32% of subjects did not reach the final assessment for the class. According to the instructor, this attrition rate is not atypical, due to the difficulties that these students have with the material. Table 1 shows the three types of attrition: early drops (students who dropped shortly after their initial assessment), in-semester drops, and drops that occurred in the last 3 weeks before the final assessment. Early drops will be excluded from analysis because the students left the class during the add-drop period, before using either condition significantly. Initial assessment scores for those who dropped out early were slightly significantly different from the full sample between conditions (*χ*^2^=3.88,p=.04887). However, among students who participated in the class, attrition rates for the experimental and control conditions were not significantly different (*χ*^2^=0.4132,p=.5203). Since only 76 learners persisted until the post-assessment, these were used to evaluate learning gains.

**Table 1:** Attrition by assigned condition

Condition	Early drops	In-semester	Finals
Exp.	7	5	11
Control	2	11	7
Total	9	16	18

### Materials

Data collection occurred through four mechanisms: ALEKS data records, the SKOPE-IT system logs, surveys on student beliefs and attitudes, and a far-transfer test on basic mathematics skills (the Basic Skills Diagnostic Test). First, the ALEKS course data included course mastery levels from adaptive assessments delivered by ALEKS (the commercial version online during the time of Fall 2014), a record of the time each student spent working in the system, and a log of student interactions with the system (e.g., right and wrong answers on each problem). These assessments impacted course grades, so students were presumably motivated to perform well. ALEKS assessments were considered the primary measure of learning gains since these are known to correlate highly with standardized tests such as the Tennessee Comprehensive Assessment program (TCAP) on similar subject matter (*r*=.842,*p*<0.01, *N*=216; Sullins et al. 2013).

Second, the SKOPE-IT system collected dialog interaction data of the student with the AutoTutor system. This included the number of inputs (student answers to AutoTutor questions), number of dialogs interacted with, number of dialogs presented (triggered, regardless of if students answered them), and LSA scores for the quality of student answers to AutoTutor.

Third, two surveys (a pre-survey and post-survey) collected student beliefs about mathematics and reactions to the tutoring agents. The pre-survey included four simple arithmetic problems (measured by speed of completion), the mathematical beliefs items from the Mathematical Problem-Solving Behaviors Scale (MPSB; Schommer-Aikins et al. 2005), and the Dweck scale for incremental and entity beliefs about intelligence (Blackwell et al. 2007). Dweck’s scale has indicated that incremental beliefs about intelligence (e.g., “I believe I can always substantially improve on my intelligence.”) tend to be associated with better learning outcomes, while entity beliefs about intelligence being fixed tend to be associated with worse outcomes (Blackwell et al. 2007). The MPSB items were collected to identify beliefs about mathematics that might influence their performance in ALEKS or interactions with the agent dialogs. The MPSB constructs are Effortful Math (EM), that math skills grow with effort, similar to Dweck’s incremental intelligence but specific to math; Useful Math (UM), that math will be valuable to them; Math Confidence (MC), their belief in being able to solve hard problems; Understand Math Concepts (UMC), that concepts rather than just finding the answer is important; Word Problems (WP), that word problems are important; and Non Prescriptive Math (NPM), that math problems are not just solved by memorizing fixed steps. Since some overlap existed between these scales, factor analysis was applied to identify factors from the pre-survey data.

In the post-survey, participants were presented with selected items regarding attitudes toward agents adapted from the Attitudes Toward Tutoring Agents Scale (ATTAS; Adcock and Eck 2005), items from the Unified Theory of Acceptance and Use of Technology (UTAUT) adapted to focus on learning while interacting with the agents (Venkatesh et al. 2003), and sections of the motivated strategies for learning questionnaire (MSLQ; Pintrich et al. 1993). Factor analysis was also anticipated for the post-survey, but low response rates made this infeasible. Six constructs from the MSLQ inventory were measured, to gather information about learners’ motivations during learning: Anxiety during testing; Time/Study Environment management and organizational habits; Effort, their self-reported time spent during the course; Peer Learning, their tendency to work with peers; Help Seeking, their tendency to ask others for help; and Metacognitive Self-Regulation, their habits for identifying gaps in knowledge and changing study habits to address them.

The UTUAT items were applied to measure Learning Expectancy (that the agents would help them learn), Effort Expectancy (that the agents were easy to work with), and Attitudes toward technology (that they like the agents in general). The ATTAS items were used to measure attitudes toward individual agents (i.e., helpfulness of the tutor versus the student), motivation to work with the agents, and technical issues (natural language smooth, feedback helpful, knew how much the user knew). The Appendix lists the items from each survey construct, along with their mean and standard deviation of responses.

Finally, a Basic Skills Diagnostic Test (BSDT) was completed by each class section in early September and again in mid-November (Epstein 2014). This test has been shown to be predictive for later success in college mathematics, so it was selected as a far-transfer test to identify potential generalized improvements to math skills. For example, AutoTutor dialogs focusing on verifying/rechecking answers might transfer to a variety of procedural problems. The BSDT is a 24-item free-response test on basic mathematics understanding. It covers skills from pre-algebra through college algebra. Of the 24 items, only nine items that aligned somewhat closely with the ALEKS items from our study. Six of the nine were fraction and decimal word problems. The remaining three aligned questions involved building and solving linear equations.

### Procedure

The experiment was conducted in three phases. First, for each class section, an in-person session was conducted that briefly explained the study, goals, and conditions. In particular, it was communicated that some might see tutoring dialogs frequently while others might see them rarely or not at all. Neither the participants nor the instructor were told which condition any particular student was part of. Following this initial orientation, students present in class were given the opportunity to complete the BSDT. The pre-survey was provided to each student was a link for a point of extra credit, with up to two reminder emails sent during the first 2 weeks of class if it was not completed. During this period, as part of their normal classroom activities, ALEKS accounts were created and some students began to use the ALEKS system. Other students dropped or transferred out of the course (i.e., the early drops).

After this first phase was complete, the SKOPE-IT system was enabled. Enabling this for the class sections meant that if a student in the experimental condition hit “Explain” on an ALEKS problem that contained a tutored explanation, they would receive dialog-based tutoring. This phase of the experiment continued until the end of the semester, with data collection occurring in ALEKS and the SKOPE-IT system. For the first 2 weeks, study personnel observed classroom use of ALEKS to identify any bugs or issues that might need to be solved. Approximately 2 weeks into this period, for approximately 3 weeks in the first half of the study, the bug in authentication allowed a subset of control condition users to encounter dialogs.

For the third phase (the last weeks of the regular semester classes), a second in-person session was conducted with each class section to administer the BSDT a second time. Students present were also given time to complete the post-survey. Students who did not attend those class sections, which was a substantial number, were reminded of the survey by email up to twice (following the same approach as the pre-survey). Attendance during these later class sections was substantially lower, due to the attrition that occurs during that course (as noted earlier). Data collection in ALEKS and SKOPE-IT continued through the remainder of the semester (approximately 2 weeks) following the final BSDT and the post-survey period.

From the instructor’s perspective, the system worked identically to the standard ALEKS system, so it was not disruptive to the standard classroom pedagogy. Completion of the surveys and BSDT test were rewarded with a minimal completion credit (e.g., one point) but, despite this, participation was inconsistent. Minor early semester technical issues were encountered due to outdated browser versions on classroom machines, but these were resolved by updating the machines.

## Results

The data collected from this study were analyzed to evaluate the impact of the SKOPE-IT system on learning gains in ALEKS, on Basic Skills Diagnostic Test outcomes, and on the relationship between students’ survey responses on their learning and behavior in the system. These results are summarized below.

### Learning gains: ALEKS assessment

Results from ALEKS assessment scores are presented in Table 2, where means are displayed and standard deviations are in parentheses. Learning gains presented are simple learning gains (i.e., post-pre) for students who completed their ALEKS final assessment. Due to random chance during assignment and early attrition, the experimental condition contained fewer subjects at both the start (*N*_*E*,0_) and end (*N*_*E*,*f*_) of the study. The experimental subjects slightly outperformed the control (+3.3 points learning gain), but this difference was not statistically significant (Cohen’s *d*=.2,*p*=.45). Attrition rates for both conditions were high (and are generally high for that course), but were not significantly different, as explained earlier in the “Participants” section.

**Table 2:** Assessment outcomes by assigned condition

Condition	Initial score (*N*_0_=103)	Final score (*N*_*f*_=76)	Learning gain (*N*_*f*_=76)	Effect size (*N*_*f*_=76)
Experimental (*N*_*E*,0_=42, *N*_*E*,*f*_=28)	20.5 (5.5)	52.6 (18.9)	31.7 (19.4)	d =2.3
Control (*N*_*C*0_=61, *N*_*C*,*f*_=48)	23.2 (7.3)	51.8 (17.1)	28.4 (15.5)	d =2.1

The dosage of AutoTutor interactions was a confound for comparing conditions. Since students took different paths through the ALEKS adaptive system, they encountered different numbers of tutoring dialogs (*M*=24 and SD=27 among students with at least one dialog). Since each example had an average of 8 dialogs, students who received dialogs saw only about 3 worked examples out of 50. Also, due to crossover issues, the “experimental” subjects only averaged four more dialogs than the “control” subjects.

To look at dose-dependent effects, a linear regression was used to model the learning gain as a function of the logarithm of the time spent in ALEKS and logarithm of the number of AutoTutor dialogs interacted with (Table 3). Logarithmic transforms were applied because diminishing learning efficiency was observed for a subset of students who overdosed on the combined system (7 students spent 80+ h in ALEKS, *σ*=1.5 above the mean). The regression improved the model fit (*R*^2^=.54) when compared to a model with only time spent studying (*R*^2^=.49). Dialog dosage was significant even after accounting for time-on-task (including time on dialogs). Including a term for dialogs that the learner encountered but ignored (e.g., returned to problem solving instead) did not improve the model fit (*t*=−.32, *p*=.75) and did not appear to be associated with greater learning.

**Table 3:** Learning by time and tutoring dialogs (*R*^2^ =.54, $R_{cv}^{2}\,=\,.54$)

Factor (*N*=76)	Coefficient (raw)	*P* value
*Log*_10_(no. of hours in ALEKS)	43.0	<.001 (t =6.6)
*Log*_10_(no. of AutoTutor dialogs interacted with)	8.0	.009 (t =2.7)
Intercept	−41.1	<.001 (t =−4.2)

### Far transfer: BSDT learning gains

Participation in the Basic Skills Diagnostic Test sessions were limited, with only 46 students completing both the pre-test and post-test administrations. Of those students who retook the test later in the semester, significant learning gains were observed, *t*(45)=2.27,*p*<.05,*d*=1.17. So then, some evidence was found that ALEKS practice improved results on the BSDT. Comparing conditions, the control condition showed a higher BSDT gain, *t*(26)=4.47,*p*<.01,*d*=1.44. Due to the low number of responses, with only 19 left in the experimental condition, caution must be taken in interpreting that result. Likewise, despite the large effect size, students’ overall improvement was limited: mean scores increased from 4.8–6.6 (out of a possible 24 items). This was primarily due to the fact that only a subset of BSDT items aligned to the course materials sequenced in ALEKS. As noted, only 9 out of 24 items aligned to the study course content.

Limiting the analysis to only the 9 aligned items, the raw gain remained small at 0.91 (from 1.57 to 2.49) but was more significant (*t*(45=4.26,*p*<.001,*d*=1.45). Gain on the aligned items did not show significant differences between conditions (*t*(42)=1.32,*p*=.19). Gain on the aligned items did correlate positively with gain on ALEKS scores (*r*(7)=.46,*p*<.001) and with time spent studying in ALEKS (*r*(7=.36,*p*<.05). However, learning gains on the BSDT test were not significantly impacted by the number of dialogs interacted with when analyzed with similar methods as for the ALEKS learning gains (i.e., no dose-dependent effects). This held true for both the full BSDT test and the aligned items. As such, analysis of BSDT results found no evidence that the dialogs caused improvements that transferred to general mathematics strategies (e.g., verifying answers).

### Attitudes toward learning, mathematics, and agents

Seventy-seven students completed the pre-survey instruments and the data were analyzed with principal component analysis using a direct oblimin rotation to obtain factors. Factor scores were computed using regression. Using the screen test, it was clear that 6 factors were good solution, accounting for 65.3% of the variance. KMO value was.761, while Bartlett’s test was significant with *p* <.0005, indicating the data is suitable for factor analysis. Components extracted were named as follows: incremental theory of intelligence (ITI, with 31.7% variance), dislike of word problems (DLW, with 9.2%), arithmetic speed (AS, with 8.2%), entity theory of intelligence (ETI, with 6.6%), effort avoidance (EA, with 5.0%), and time persistence (TP, with 4.7%). All component factor score correlations were low, however. In this data, ITI was correlated negatively with ETI (*r*(75)=−.254,*p*=.026), negatively with EA (*r*(75)=−.253,*p*=.026), and positively with TP (*r*(75)=.258,*p*=.024).

Further, we examine the relationships of these factors in students with important dependent measures we collected. In some cases, attrition resulted in reduced *df*s for the comparison, which is noted. For the first ALEKS assessment, only a correlation with AS was significant (*r*(63)=.254,*p*=.026). For the ALEKS last assessment, most interesting in these results were the correlations with EA, which was composed of 4 negatively weighted MPSB items for items such as “Working can improve one’s ability in mathematics” and similar items that correspond to a domain (math) specific form of incremental theory of intelligence (like the ITI factor, but for math). For ALEKS last assessment, ALEKS gain, and total time in the system were negatively correlated with EA (*r*(63)=−.309,*p*=.012;*r*(63)=−.303,*p*=.014;*r*(63)=−.262,*p*=.021, respectively). Similarly, we saw negative correlation between ETI and total time in the system (*r*(63)=−.238,*p*=.037). Finally, we also saw a correlation between ALEKS learning efficiency and the EA component (*r*(63)=.263,*p*=.034).

Participation of students was lower for the post-survey, despite the same completion credit as for the first survey (*N* = 43). This small sample prohibited factor analysis, so we used the pre-planned question categories to describe the relationships to dependent measures. First, for the peer learning component of the MSLQ, we saw negative correlations with the last ALEKS assessment and ALEKS gain, (*r*(41)=−.308,*p*=.044;*r*(41)=−.334,*p*=.029). Next, we saw a substantial relationship between the MSLQ component for time/study environmental management, with positive correlation with the last ALEKS assessment, ALEKS gain, and total time in the system (*r*(41)=.453,*p*=.002;*r*(41)=.516,*p*<.0005;*r*(41)=.580,*p*<.0005). These correlations seem to indicate the importance of intentional study and are similar to the negative correlations for EA noted previously.

**Fig. 6:**
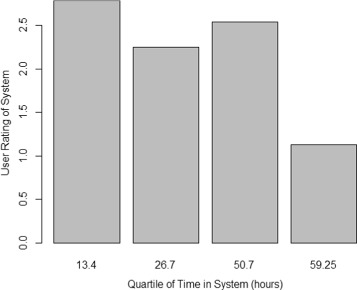
User attitude toward system by quartile of time spent in ALEKS

For the technology attitude items, all three factors related to agents (overall attitude, learning expectancy, and effort expectancy) were highly correlated (*r*(41)=.853 to *r*(41)=.972; *p*<.01 for all). As such, overall impressions of the tutoring system were strongly connected. We saw negative correlations with total time in the system, which reached significance for overall attitude about the system and learning expectancy from the system (*r*(41)=−.331,*p*=.030;*r*(41)=−.330,*p*=.031). However, as shown in Fig. 6, this drop in attitudes toward the system was not uniform and was driven by the quartile of post-survey respondents who used the system significantly longer than the average. We also saw that learning expectancy correlated positively with the average LSA match score for students who received SKOs (*r*(21)=.413,*p*=.050), indicating that students who did well on the dialogs felt that they learned more from them. Finally, we observed a trend between incremental theories of intelligence (ITI) and all three technological acceptance measures with all *p*s <.11, with an average *r* of.29.

## Discussion and conclusions

Due to insufficient differences in dosage, the experimental and control conditions showed no significant differences in learning gains. With that said, the dosage of tutoring dialogs was significantly associated with learning gains (*p*<.01). Moreover, no other explanatory factor was found that captured this difference. Student prior knowledge did not correlate with dialog interaction (*r*=−.03, *p*=.39). Also, dialogs were only associated with learning when the learner interacted with them, making it unlikely that higher-achieving students simply encountered more dialogs. Students were also unaware of which topics had dialogs, so there was little likelihood that higher-achieving learners self-selected opportunities to use the dialogs. Finally, regression analyses found that AutoTutor dialogs predicted learning even after accounting for total time studying in the combined system. As such, a preponderance of evidence weighs toward the hypothesis that AutoTutor dialogs improved learning gains as compared to ALEKS alone (H1), but the crossover effects of dialogs in the Control condition undermined the randomized assignment that would have provided causal evidence to this effect.

Results of the Basic Skills Diagnostic Test showed that BSDT items aligned to the ALEKS experimental content improved, but that these gains were only impacted by time spent in ALEKS and did not appear to be affected by the dialogs completed. The purpose of this far-transfer test was to determine if certain generalizable skills and strategies from the dialogs (e.g., identifying variables, verifying answers) would improve performance on related general mathematics tasks. At least with the level of dosage provided in this study (approximately 1 h of dialogs), students’ approach to mathematics on the BSDT did not lead to systematically higher math performance. As such, the second hypothesis (H2) was not confirmed.

One issue on which this study sheds light was some individual differences from the surveys that reflected different outcomes in ALEKS. A second key issue is which parts of the SKOPE-IT explanation promote learning in this context. Compared to the standard ALEKS system, SKOPE-IT added three new elements: (A) Isomorphic worked examples presented at an impasse point, (B) animated agents, and (C) tutored self-explanations. Of these three factors, based on prior research with AutoTutor, we suspect that the tutored self-explanations primarily controlled any learning gains (Graesser et al. 2012). Insights about each component will be discussed briefly below.

### Individual differences in performance

Survey results indicated that certain students benefited more from the ALEKS system overall, but there were no clear indications that certain types of students benefited more from the dialogs. While participants’ initial knowledge only correlated with their arithmetic speed, their learning gains and final assessment scores were associated with math-specific views about the value of effort (math effort avoidance; EA) and self-reported study habits (MSLQ time and study environment). The EA (reverse coded) may indicate a math-specific form of incremental theory of intelligence that helped certain students be more persistent. These beliefs predicted greater amounts of time spent studying in ALEKS and increasing learning due to time-on-task. However, those beliefs did not mean those students learned at a faster rate, since avoiding effort was associated with higher learning efficiency, possibly due to students avoiding or quitting harder or more time-consuming problems.

The negative correlation between preferences toward peer learning and learning gains was unexpected and open to interpretation. The course format for the study included little peer-group work since it primarily used lectures and ALEKS practice. One possible hypothesis is that certain students genuinely learn more effectively in small-group contexts and were able to accurately report that. However, there seems to be little theory that would support this hypothesis. Alternatively, personality traits such as extroversion might not only lead to a preference to peer studying but also more socialization (leading to less studying overall), though this was not demonstrated in their time spent studying in ALEKS (which was not correlated with self-reported peer learning patterns). More likely, since the MSLQ was administered toward the end of the course, students who were struggling might have reported more need for peer support, which could explain the negative correlation with their final assessments.

### Isomorphic worked examples

The contribution isomorphic worked examples after an impasse appeared to have mixed results. Based on student exit-survey comments and classroom observations, learners often complained that “The system didn’t explain the problem that I was working on.” While the students were instructed that the tutoring dialogs worked through a problem that had exactly the same steps (only different numbers and variable names), they struggled to connect the tutored explanation to their original impasse. This may indicate that parts of this student population was focused on procedural fluency (e.g., diagnosing calculation errors) rather than conceptual fluency (e.g., understanding the principle that was needed). While this may not impact learning directly, it could have impacted students’ engagement when working with the agents.

This interpretation might be supported by further data since positive perceptions of agents showed a trend with ITI beliefs (which, if maintaining the same correlation level, would have been significant with a higher turnout for the post-survey). If this correlation reached significance, it would support the hypothesis that learners would prefer agent dialogs if they were interested in conceptual mastery (H3). However, our results do not entirely support this hypothesis. Even if this effect reached significance, the overall magnitude would be lower than anticipated (approximately *r*=.3) which indicates that a variety of other factors impact perceptions of the tutoring dialogs and agents.

Instead, mixed receptions to the system are also likely due to implementation details that may have reduced its effectiveness. One problem with the study implementation might have been due to hiding future steps of the worked example when performing the tutoring. In this study, SKOPE-IT pages were sequenced one step at a time to help scaffold learners thinking through the problem (intended to reduce cognitive load). However, multiple survey responses indicated that they preferred to see the whole worked example first, as was done in normal ALEKS explanations. Students might have been better able to self-explain after reviewing processes shown in the example so that dialogs are used to reflect on the steps after seeing the full example (Schworm and Renkl 2007). This would be particularly relevant for problems with interdependencies between steps or skills. While it had originally been thought that addressing one step at a time represented the most scaffolded form of presenting a worked example, it might instead be that presenting the full-worked example as a mental model up-front is a prerequisite for effective dialog scaffolding and that a linear step-by-step progression represents a harder, more faded task (Atkinson et al. 2003).

From the standpoint of designing future systems, rather than giving a fully tutored example, this population of students may prefer a system where they could select one or two steps to complete tutoring dialogs after so that they could focus on solidifying the specific concepts they were struggling with at that moment (i.e., targeting their own top impasses). This approach might have also allowed expanding the coverage of ALEKS topics: as noted, only 30 out of 240 course topics were covered (and some were ones students never reached). In general, for aligning a smaller adaptive system to a larger one, a broader and limited intervention may be more predictable for dosage (e.g., one or two dialogs per example type, as opposed to 5–12 for full coverage of an example).

A second potential improvement would be enabling fast inquiry for student questions. Asking questions of the agents about steps was also requested by students in the post-survey open-response answers. This functionality was not implemented in this version of the system but could be added to future versions. One effective methodology for such questions might be the point-and-query interface studied by Graesser et al. (1992), which allows clicking on elements to see a list of questions and check answers quickly.

### Animated agents

Reactions to the animated agents were mixed to negative. While some students found the agents engaging, other students complained about the relative speed of working with the agents versus reviewing the standard ALEKS worked examples. Negative sentiments were more common among users who spent longest (top quartile) in the ALEKS system mastering topics. Looking at the open-response answers of users who used the ALEKS extensively and rated the agents lowest, 50% of these responses focused on either time spent working with the agents or a preference for text. A subset of responses also noted that the animated agents were slower than a text-only interface, which was available as an alternate dialog mode.

Overall, reactions to the agents were almost unidimensional: students who liked the agents (overall attitude) felt that they learned from the dialogs (learning expectancy) and that the agents were easy to interact with (effort expectancy). Given the very high correlations between these factors, as well as the fact that students who gave better answers (higher LSA scores) expressed a higher learning expectancy from the agents, students may also be estimating their learning and their opinions of the agents based on how well they could answer the agents’ questions. This may indicate that students like or dislike the agents based on the amount of effort they needed to apply to complete dialogs. This issue would be related to the tendency for many students to dislike learning tasks that they find cognitively demanding (Willingham 2009). Unfortunately, many productive learning tasks are not easy or well liked. While the AutoTutor dialogs were not more challenging than solving the ALEKS math problems, learners needed to reflect on the problem to answer correctly, rather than only manipulate terms to try to reach an answer. As such, some students may have found the dialogs more challenging or less intuitive, particularly since many US students are seldom taught to talk about math.

Prior findings with AutoTutor indicated little additional learning gains from using animated agents as opposed to voice only or from even text-only interactions (Craig et al. 2004; Nye et al. 2014a). General reviews of pedagogical agents have likewise found either small effects on learning (Schroeder et al. 2013) or no effects on learning (Heidig and Clarebout 2011) due to animated agents, which indicates that pedagogical agents may only be useful in certain contexts. Some design advantages for agents appear to be allowing users to control pacing, using agents to deliver explanations (as opposed to only feedback), and using voice delivery rather than text (Heidig and Clarebout 2011). In this study, the agents worked with students to self-explain, used voice delivery by default, and provided a faster text-only option for limited control over pacing. Unfortunately, voice delivery appears to conflict with pacing: voice was viewed as slow by most users (though one felt it was too fast), so text with no agent may potentially be preferred by many users. Given this background, the faster text-only mode may be the preferred default for integrating with a system such as ALEKS, due to the noticeable difference in pacing (e.g., very quick reviews of worked examples versus thorough dialogs about concepts) and negative responses to surface-level details such as the animations or voices of the agents. This also raises the issue that the interactions with an agent should match its surrounding environment and expectations: faster agents for faster-paced environments or explicit self-pacing may be important.

### Tutored self-explanations

The conversational tutoring itself (as opposed to the agents) was less controversial, and a number of students requested additional features (e.g., answering the learner’s questions). However, evident in the post-survey open responses, a subset of learners did not understand how talking about math concepts would help them improve at math. The general theme of these comments was that the tutoring did not show them “how to get the correct answer.” This line of thought implies that getting the right answer to the current problem is equivalent to learning (e.g., number of problems solved might be their internal metric for mastery). This belief is likely reinforced in some areas of the math curriculum, where drill-and-practice approaches can be an efficient way to master simple calculations (e.g., multiplication tables) and by the grading practices of teachers who may rarely assign conceptual exercises, but rather assign computational problem sets, which students must answer correctly to receive high grades.

Despite the perceptions of some students, prior research on this topic has found that self-explanation prompts do produce deeper understanding, such as identifying when a problem cannot be solved. Aleven and Koedinger (2002) also found that while students spent more time per example, overall learning efficiency was the same (i.e., students gained more per example). Follow-up work on this topic found that switching from menu-based explanation choices to natural language choices can also improve explanation skills (Aleven et al. 2004). Unfortunately, many students do not intuitively understand that self-explanation can improve the efficiency of their learning (i.e., save them time in the long run). Students may need domain-specific metacognitive training to understand the value of self-explanation and tutoring dialogs.

## Conclusions and future directions

In terms of the major research questions approached by this study, the first hypothesis (tutoring dialogs improve learning gains) had moderate but not conclusive support, the second hypothesis (dialogs will transfer to general mathematics competency) was not supported by this study, and the third hypothesis (student beliefs about math concepts and learning influence perceptions of agents and tutoring) had insufficient support but showed a trend that might be confirmed if more data were collected. Additionally, a number of directions for future work were uncovered. First, student reactions to the system were mixed to negative. One primary problem was that while the student was at an impasse, they could not easily relate the isomorphic problem to their impasse. This implies that students may benefit from starting with tutored worked examples integrated as a supplementary activity, before they are integrated into a problem-solving impasse, to help students become familiar with a dialog-based pedagogical approach. Alternatively, it may indicate that tutoring for worked examples is most effective when the whole worked example is presented first and then student supported to self-select one or more dialogs that match their current impasse.

Second, some students’ inability to generalize their improved knowledge from ALEKS practice back to the BSDT skills indicated the need to explicitly train students on the role and importance of self-explanation, identifying problem types, and other domain-specific cognitive and metacognitive strategies. Prior work such as Chi and VanLehn (2010) has showed that tutoring domain-specific problem-solving strategies can produce significant learning gains, particularly for low-performing learners. However, particularly among low-performing students, it may be necessary to clearly and explicitly link these strategies to their performance. Some potential methods to approach this could be graded activities on using the strategies (rather than only interleaving them into examples) or demonstrating how they benefit procedural fluency (e.g., presenting problem types where recognizing and using such strategies are particularly important).

While this research has raised many questions, it has also provided key insights for designing and studying adaptive learning systems. Findings from the MSLQ showed the importance of self-regulating study habits, which were significantly associated with overall learning gains. Likewise, effort avoidance for math was associated with worse overall learning but greater efficiency, indicating that such a scale might be adapted to help predict when students are likely to disengage from an adaptive system based on diminishing returns. Related to this, models of mastery gains in ALEKS showed an overall decrease in gains as a function of time. In principle, this was not necessarily expected: adaptive learning systems such as ALEKS are designed to try to break down skills so that all prerequisites are already known, so later topics are not necessarily harder. However, assuming that individuals switch away from harder topics that they do not master, it is reasonable to assume that learners in a self-directed system such as ALEKS will eventually complete most of the “low-hanging fruit” and be left with a fringe of challenging topics that slow down progress. Together with surveys that help determine effort avoidance, such system-wide learning curves might help determine when students may need a human intervention to avoid disengagement. Such work would provide a larger framework for considering wheel spinning (Beck and Gong 2013), but considering the system level rather than an individual topic.

New findings about dialog systems and agents were also uncovered, though as even relatively recent meta-analyses such as Heidig and Clarebout (2011) note, research on pedagogical agents has only a limited number of first principles. Our study findings indicate tradeoffs even between some of the well-established principles such as interactivity, control over pacing, and voice (Chi 2009; Heidig and Clarebout 2011). In particular, voice and interactivity have an inherent minimum pacing that is slower than skimming text or skipping through to useful parts of a video. We also found that, for this type of population at least, survey ratings about pedagogical agents tended to be highly univariate (i.e., one global “liking” factor appears to drive most of the variance). This indicates that alternate approaches such as open-response questions, A/B comparisons, storyboards, or interviews might be preferred over detailed survey inventories. In particular, topic analysis of open-response questions might be valuable: while the Likert survey answers were quite univariate, the open-response questions showed clear actionable themes that could likely be aligned to a frameworks such as the Pedagogical Agents–Conditions of Use Model (PACU) and Pedagogical Agents–Levels of Design (PALD; Heidig and Clarebout 2011).

Finally, the positive correlation between answering the tutor correctly and liking to use the tutors indicates a potentially difficult balance between encouraging engagement with ITS versus providing optimal challenge levels (Willingham 2009). Game design principles for modulating challenge levels may be required (e.g., mixtures of easy and hard progressions). This becomes increasingly complicated as we integrate multiple intelligent systems, which use different interaction and pacing strategies. Guidelines for providing continuity, engagement, and managing learner expectations will all be key future areas to study as we begin further work on meta-adaptive systems that switch between different intelligent learning environments (Nye 2016).

## Appendix

### Summary of survey responses and full ALEKS worked example

Note: Certain items may be modified slightly from their original form, in order to help participants understand what was being referred to (e.g., the system versus the animated tutors).

**Table 4:** Pre-survey item means and standard deviations

Construct	Item text	Mean	St. dev.
Dweck entity	I don’t think I personally can do much to increase my intelligence.	1.76	1.13
Theory of intel.	My intelligence is something about me that I personally can’t change very much.	2.15	1.33
	To be honest, I don’t think I can really change how intelligent I am.	2.15	1.45
	I can learn new things, but I don’t have the ability to change my basic intelligence.	2.15	1.33
Dweck incremental	With enough time and effort I think I could significantly improve my intelligence level.	5.12	1.21
Theory of intel.	I believe I can always substantially improve on my intelligence.	5.1	1.23
	Regardless of my current intelligence level, I think I have the capacity to change it quite a bit.	5.1	0.99
	I believe I have the ability to change my basic intelligence level considerable over time.	5	1.1
MPSB math	I feel I can do math problems that take a long time to complete.	4.01	1.36
confidence	Math problems that take a long time don’t bother me.	2.88	1.48
	I’m not very good at solving math problems that take a while to figure out.	3.63	1.42
MPSB Effortful	Ability in math increases when one studies hard.	4.87	1.21
Math	By trying harder, one can become smarter in math.	5.06	1.14
	I can get smarter in math if I try hard.	4.95	1.16
	Working can improve one’s ability in mathematics.	4.74	1.33
	I can get smarter in math by trying harder.	5.08	1.1
	Hard work can increase one’s ability to do math.	5.03	1.14
	I find I can do hard math problems if I just hang in there.	4.26	1.41
MPSB Non-	Any word problem can be solved if you know the right steps to follow.	5.03	1.23
Prescriptive	Any word problem can be solved by using the correct step-by-step procedure.	4.91	1.3
MPSB persistence	If I can’t do a math problem in a few minutes, I can’t do it at all.	2.59	1.32
	If I can’t solve a math problem quickly, I quit trying.	2.54	1.37
MPSB understand	It’s not important to understand why a mathematical procedure works as long as it gives a correct answer.	2.24	1.51
MPSB concepts	Getting the right answer in math is more important than understanding why the answer works.	2.58	1.49
	It doesn’t really matter if you understand a math problem if you can get the right answer.	2.22	1.28
MPSB Useful Math	Studying mathematics is a waste of time.	2.28	1.44
	Mathematics has no relevance to my life.	2.44	1.48
	Mathematics will not be important to me in my life’s work.	2.6	1.58
	Knowing mathematics will help me earn a living.	4.55	1.36
	Mathematics is a worthwhile and necessary subject.	4.54	1.36
	I study mathematics because I know how useful it is.	3.91	1.4
MPSB Word	Word problems are not a very important part of mathematics.	2.56	1.37
Problems	Math classes should not emphasize word problems.	3.29	1.49

(*N* = 77; scale: 1-strongly disagree to 6-strongly agree)

**Table 5:** Post-survey item means and standard deviations

Construct	Item text	Mean	St. dev.
MSLQ Anxiety	When I take a test I think about how poorly I am doing compared with other	4.23	1.75
	students.		
	When I take a test I think about items on other parts of the test I can’t answer.	4.16	1.45
	When I take tests I think of the consequences of failing.	4.86	1.44
	I have an uneasy, upset feeling when I take an exam.	4.02	1.39
	I feel my heart beating fast when I take an exam.	4	1.56
MSLQ Effort	Even when course materials are dull and uninteresting, I manage to keep working	4.47	1.09
	until I finish.		
MSLQ Help Seeking	Even if I have trouble learning the material in math class, I try to do the work on	4.72	1.17
	my own, without help from anyone.		
	I ask an instructor to clarify concepts I don’t understand well.	3.88	1.33
	When I can’t understand the material in a math class, I ask another student in	3.47	1.45
	class for help.		
MSLQ Metacognitive	During class time I often miss important points because I’m thinking of other things.	3.51	1.28
Self-Regulation	When reading for math class, I make up questions to help focus my reading.	3.37	1.45
	When I become confused about something I’m reading for math class, I go back	4.67	1.34
	and try to figure it out.		
	If course readings are difficult to understand, I change the way I read the material.	3.91	1.03
	Before I study new course material thoroughly, I often skim it to see how it is	4.26	1.18
	organized.		
	I ask myself questions to make sure I understand the material I have been studying	4.09	1.34
	in math class.		
	I try to change the way I study in order to fit the course requirements and the	3.84	0.99
	instructor’s teaching style.		
	I often find that I have been reading for math class but don’t know what it was all	4.09	1.2
	about.		
	I try to think through a topic and decide what I am supposed to learn from it rather	4.07	1.35
	than just reading it over when studying for a math class.		
	When studying for this course I try to determine which concepts I don’t understand well.	4.63	1.12
	When I study for math class, I set goals for myself in order to direct my activities in each	4.02	1.19
	study session.		
	If I get confused taking notes in class, I make sure I sort it out afterwards.	3.95	1.54
	When studying for math class I try to determine which concepts I don’t understand well.	4.74	1.01
MSLQ Peer Learning	When studying for math class, I often try to explain the material to a classmate or friend.	3.02	1.64
	I try to work with other students from math class to complete the course assignments.	3.05	1.35
MSLQ Time/Study	I usually study in a place where I can concentrate on my course work.	4.79	1.19
Environmental	I make good use of my study time for math class.	4.12	1.26
Management	I find it hard to stick to a study schedule.	3.98	1.39
	I have a regular place set aside for studying.	4.35	1.38
	I make sure that I keep up with the weekly readings and assignments for math class.	4.33	1.03
	When taking a math class, I attend class regularly.	5.09	1.03
	I often find that I don’t spend very much time on math classwork because of other activities.	3.81	1.33
	I rarely find time to review my notes or readings before an exam.	2.98	1.36
ATTAS attitude	Working with the tutoring system was fun.	2.23	1.39
	I liked working with the tutoring system.	2.23	1.43
	I think using the tutoring system is a good idea.	2.63	1.6
	The tutoring system could make learning more interesting.	2.67	1.47
UTUAT effort	The tutoring system makes sense and I could understand it.	2.56	1.42
expectancy	I found the tutoring system easy to use.	2.7	1.56
UTUAT learning	I found the tutoring system useful for my learning.	2.42	1.42
expectancy	Using the tutoring system could help me learn more quickly.	2.33	1.44
	Using the tutoring system could increase how much studying I could get done.	2.53	1.5
	I think the tutoring system could help me learn difficult math concepts.	2.74	1.54
	The tutoring system helped me more than I expected.	2.28	1.34

(*N* = 43; scale: 1-strongly disagree to 6-strongly agree)

**Fig. 7:**
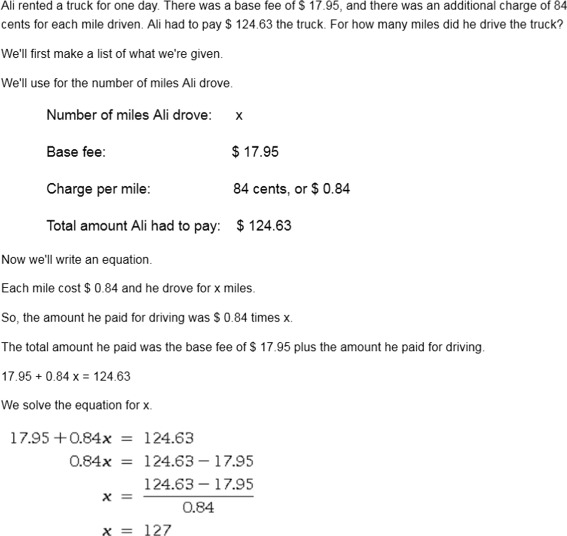
Full-worked example for tutored example shown in Fig. 4

Note: This is one of the longest worked examples tutored, so it can be considered the maximum size for a worked example in SKOPE-IT.
